# OC-2-KB: integrating crowdsourcing into an obesity and cancer knowledge base curation system

**DOI:** 10.1186/s12911-018-0635-5

**Published:** 2018-07-23

**Authors:** Juan Antonio Lossio-Ventura, William Hogan, François Modave, Yi Guo, Zhe He, Xi Yang, Hansi Zhang, Jiang Bian

**Affiliations:** 1 Health Outcomes & Biomedical Informatics, College of Medicine, University of Florida, 2004 Mowry Road, Gainesville, FL, 32610 USA; 2 School of Information, Florida State University, 142 Collegiate Loop, Tallahassee, FL, 32306 USA

**Keywords:** Semantic web knowledge base, Information extraction, Biomedical named-entity recognition, Relation extraction, Crowdsourcing, Obesity, Cancer

## Abstract

**Background:**

There is strong scientific evidence linking obesity and overweight to the risk of various cancers and to cancer survivorship. Nevertheless, the existing online information about the relationship between obesity and cancer is poorly organized, not evidenced-based, of poor quality, and confusing to health information consumers. A formal knowledge representation such as a Semantic Web knowledge base (KB) can help better organize and deliver quality health information. We previously presented the OC-2-KB (Obesity and Cancer to Knowledge Base), a software pipeline that can automatically build an obesity and cancer KB from scientific literature. In this work, we investigated crowdsourcing strategies to increase the number of ground truth annotations and improve the quality of the KB.

**Methods:**

We developed a new release of the OC-2-KB system addressing key challenges in automatic KB construction. OC-2-KB automatically extracts semantic triples in the form of subject-predicate-object expressions from PubMed abstracts related to the obesity and cancer literature. The accuracy of the facts extracted from scientific literature heavily relies on both the quantity and quality of the available ground truth triples. Thus, we incorporated a crowdsourcing process to improve the quality of the KB.

**Results:**

We conducted two rounds of crowdsourcing experiments using a new corpus with 82 obesity and cancer-related PubMed abstracts. We demonstrated that crowdsourcing is indeed a low-cost mechanism to collect labeled data from non-expert laypeople. Even though individual layperson might not offer reliable answers, the collective wisdom of the crowd is comparable to expert opinions. We also retrained the relation detection machine learning models in OC-2-KB using the crowd annotated data and evaluated the content of the curated KB with a set of competency questions. Our evaluation showed improved performance of the underlying relation detection model in comparison to the baseline OC-2-KB.

**Conclusions:**

We presented a new version of OC-2-KB, a system that automatically builds an evidence-based obesity and cancer KB from scientific literature. Our KB construction framework integrated automatic information extraction with crowdsourcing techniques to verify the extracted knowledge. Our ultimate goal is a paradigm shift in how the general public access, read, digest, and use online health information.

## Background

Overweight and obesity are associated with 2.8 million deaths throughout the world. More than 1.9 billion adults (39% of adults) were overweight in 2014; of which, over 600 million (13% of adults) were obese. More than one-third (34.9% or 78.6 million) of US adults are obese and the prevalence of obesity among children is also increasing [1]. As a result of many meta- and pooled analyses on obesity-related cancers, existing knowledge and data on obesity and the increased risk it poses for cancer, are proliferating. Obesity and overweight are related with major risk factors for cancers of the endometrium, breast, kidney, colorectal, pancreas, esophagus, ovaries, gallbladder, thyroid, and possibly prostate [2–5]. Obesity accounts for 3–10% of cancer cases and deaths [6, 7], and it will be linked to more cancer cases than smoking within ten years [4].

On the other hand, interventions that may effectively modify excess weight can be used for cancer control, preventing further cancer burden [8]. Many health behavior theories, such as the information-motivation-behavioral skills mode (IMB) [9] and the integrated behavior model (IBM) [10], recognized that an individual needs the information and knowledge to initiate and perform health behavior changes towards healthier lifestyles. Increasingly, people engage in health information seeking via the Internet. In the US, 87% of adults have Internet access and 72% look online for health information [11]. Meanwhile, the quality of online health information varies widely [12–14]. In particular, existing online information on obesity, especially its relationship to cancer, is heterogeneous ranging from pre-clinical models to case studies to mere hypothesis-based arguments. While the direct causal relationship between obesity and cancer has been difficult to definitively prove, research studies have generated a tremendous amount of supporting data. But collectively, these data are poorly organized in the public domain. Typical consumers cannot translate the vast amounts of online health information into usable knowledge nor can they assess its quality. Online health information consumers face a number of access barriers including information overload and disorganization, terminology and language barriers, lack of user friendliness, and inconsistent updates [13, 15, 16].

There is an urgent need to organize the vast and increasing amount of information on possible links between obesity and cancer in a way that helps consumers understand and use the information in a meaningful way. This involves collating the information, linking it to evidence in the scientific literature, evaluating its quality, and presenting high quality information relevant to their specific questions in a user-friendly way. Thus, we seek to fill critical gaps in knowledge representation of obesity and its relationship to cancer, improve dissemination of knowledge in obesity and cancer research, and ultimately to provide the general public with a knowledge base (KB) of well-organized obesity and cancer information to help them make informed health decisions.

Creating KBs has been an active research area with academic projects such as YAGO [17] and NELL [18], community-driven efforts such as Freebase [19] and Wikidata [20], and commercial projects such as those by Google [21] and Facebook [22]. All of these KBs used a formal ontology-driven knowledge representation, using the Resource Description Framework (RDF) and the Web Ontology Language (OWL) to form the Semantic Web. Regardless of the debate on what is an ontology [23], an ontology can be used to encode the knowledge (i.e., specifically, the logical structure) of a domain at the semantic-level under a controlled, standardized vocabulary for describing entities and the semantic relationships between them. For example, a statement “obesity is a risk factor for cancer.” can be decomposed into a semantic triple, where the relationship between two entities“obesity” and “cancer” is “is a risk factor for”. An ontology-driven knowledge base provides a consistent representation of the facts about the domain and makes it possible to reason about those facts and use rules or other forms of logic to deduce new facts or highlight inconsistencies [24].

In the biomedical domain, there also have been several efforts in making Semantic Web KBs, such as the “Gene Ontology knowledgebase” [25, 26], UniProt [27], Literome [28], BioNELL [29], and SemMedDB [30]. These KBs provided a huge collection of entities and facts, but nonetheless, contained noises and can be unreliable due to the limited accuracy of their information extraction (IE) systems. For example, similar to our effort, SemMedDB was created with SemRep [31] based on the Unified Medical Language System (UMLS) to extract semantic triples. As of June, 2017, SemMedDB contained 91.6 million triples extracted from 27.2 million PubMed citations. Nevertheless, unlike SemRep, our approach can accurately identify meaningful entities beyond the limitation of having to be in existing terminologies. Further, recent studies have reported numerous quality issues (e.g., inaccurate facts) in the SemMedDB [32–34], for instance, “Insuli-AUGMENTS-catecholamine secretion” [35]. A major challenge in KB construction is to assess the quality of the candidate facts extracted by the IE system, and turn them into useful knowledge [36].

Crowdsourcing is a way to outsource a task to a group or community of people [37]. Crowdsourcing allows to annotate enormous volumes of data in a short time, and opened a new door to tackle complex problems and challenges in research [38, 39]. KBs can also be curated via crowdsourcing. Wikipedia (although not a Semantic Web KB) is the classic example of massively decentralized, peer-produced KB on the Web. In terms of Semantic Web KBs, Freebase [40] and Wikidata [41] are two successful crowd-sourced examples in the general domain. The use of crowdsourcing to solve important problems in biomedical domain is also growing and has a wide variety of applications [42]. Relevant to this project, crowdsourcing is successful in biomedical natural language processing (NLP) especially on named-entity recognition [43–45], curating clinical ontology [46, 47], constructing specialized biomedical KBs [48, 49].

In our previous work [50], we created a IE software pipeline, Obesity and Cancer to Knowledge Base (OC-2-KB), aiming to build an evidence-based obesity and cancer Semantic Web KB. Using a set of NLP and machine learning techniques [51], OC-2-KB was able to automatically extract subject-predicate-object semantic triples from PubMed abstracts related to obesity and cancer. In this paper, we present a new release of the OC-2-KB system (available at: https://github.com/bianjiang/BioText-2-KB), and investigated the use of crowdsourcing to improve the quality of the extracted facts. Further, the crowd-sourced semantic triples are fed back to the machine learning modules to enrich the training corpus and subsequently improve the performance of the IE system.

The primary contributions of this work in comparison to our previous study [50], are detailed below:

- We conducted two rounds of crowdsourcing experiments to assess the feasibility of adding human knowledge to the KB construction process.
- We extended our original corpus from 23 random obesity and cancer related Pubmed abstracts to 82 abstracts from systematic review papers. We curated a preliminary obesity and cancer knowledge base (OCKB) with these abstracts using OC-2-KB and evaluated the query results against this OCKB.
- Finally, we refined the underlying machine learning models incorporating the crowdsourcing results.

The rest of the paper is organized as follows. We will briefly recap the OC-2-KB framework, and the crowdsourcing approach in the “Methods” section. The results of the crowdsourcing experiments and our evaluations of the updated machine learning models will be presented in the “Results” section. We will discuss the lessons learned, conclude the current work, and present future directions in the “Discussion and Conclusions” section.

## Methods

### The obesity and cancer to knowledge base (OC-2-KB) information extraction pipeline

**Fig. 1:**
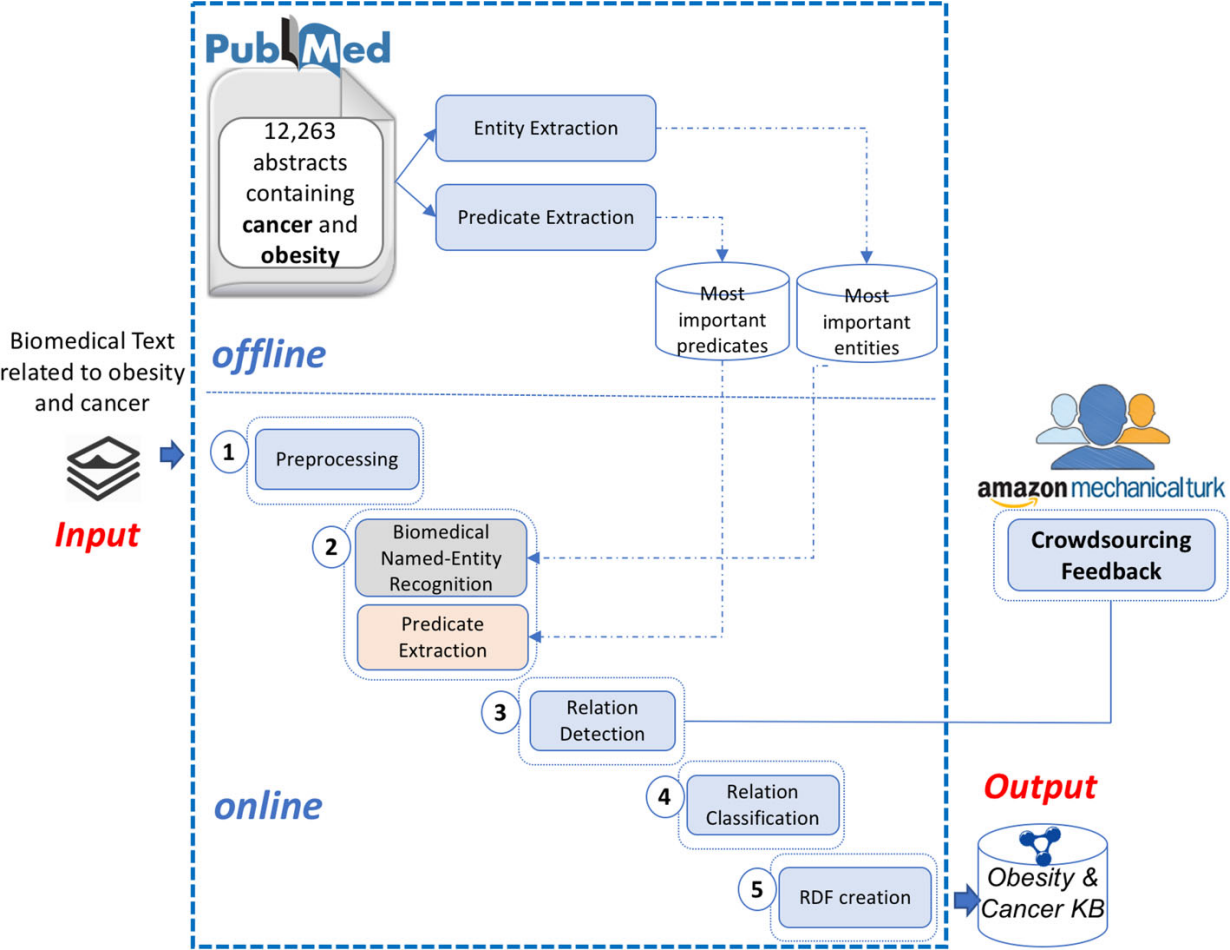
An overview of the new OC-2-KB information extraction system with crowdsourcing feedback. The *offline* module creates dictionaries of domain-relevant entities and predicates. The *online* module extracts facts from scientific literature to construct an obesity and cancer knowledge base

Figure 1 shows the overview of the new release of OC-2-KB, where we added a new step called “Crowdsourcing Feedback” to the original OC-2-KB system. OC-2-KB has two components: (1) an offline process that identifies the most domain-relevant entities and predicates with importance scores, and (2) an online process that can automatically extract the facts in the format of semantic triples from relevant scientific text. For the sake of completeness, we briefly describe the crucial components of the OC-2-KB system along with our new experiments. Please refer to [50] for more system details.

#### The offline process: identify domain-relevant entities and predicates

The offline process was part of the configuration process of the OC-2-KB system. As our goal is to create a KB related to obesity and cancer, we used the titles and abstracts of PubMed articles containing the keywords “obesity” and “cancer” to create the dictionaries. A total of 12,263 articles were extracted. We used the *LIDF-value* [52] measure implemented in *BioTex* [53] to assess the importance of the candidate entities and predicates. Two separate dictionaries were created, which contained approximately 34,500 entities and 8,200 predicates, respectively. The entity and predicate dictionaries were then be used as inputs to the biomedical named-entity recognition (BioNER) and predicate extraction process as described below.

#### The online process: extract semantic triples from scientific literature

The online process has 5 main steps to extract facts in the format of semantic triples from scientific literature related to obesity and cancer.

- ***Input:***PubMed titles and abstracts relevant to obesity and cancer.
- **Step 1 - Preprocessing:** Using the Stanford CoreNLP toolkit, we first preprocessed the PubMed titles and abstracts to split each document into a collection of sentences (i.e., sentence segmentation) as the semantic triples are currently extracted from each sentence independently.
- **Step 2 - Biomedical Named-Entity Recognition (BioNER) and Predicate Extraction:** Based on the entity and predicate dictionaries created in the offline process, we then extracted candidate entities and predicates from each sentence. As discussed in our previous work [51], our methods for biomedical NER and predicate extraction were based on both linguistic and statistic features of the text, and outperformed other state-of-the-art systems on our obesity and cancer corpus. Our experiments obtained *F-measures* of 90.1 and 51.8% for entity and predicate extractions, respectively.
- **Step 3 - Relation Detection:** Using supervised classifiers with a set of statistical, lexical, and semantic features [51], we were able to determine whether a pair of two candidate biomedical entities and a candidate predicate formed a valid assertion as a subject-predicate-object statement. The random forest algorithm achieved the best performance with an *F-measure* of 84.7% on an independent test set.
- **Step 4 - Relation Classification:** After relation detection, we normalized the predicate to one of thirteen relation classes using a multi-class classifier. Based on a manually annotated sample corpus (i.e., 23 obesity and cancer abstracts), we previously adopted [50] 12 relation classes from the Relation Ontology (RO) [54]. We added an “other” category in this work to temporarily hold all predicates that cannot be classified into one of the 12 specific relation classes. With the same set of statistical, lexical, and semantic features [51], the random forest model achieved the best results with an *F-measure* of 85.3% on a independent test set.
- **Step 5 - RDF Graph Creation:** The semantic triples extracted in the previous steps were then inserted into a graph database (i.e., GraphDB [55]) using the RDF4J [56] framework.

### Incorporating crowdsourcing feedback

Even though our machine learning models demonstrated state-of-the-art performance, they were not perfect. Thus, we investigated a crowdsourcing strategy to validate the extracted semantic triples. For this purpose, we used Amazon Mechanical Turk (MTurk) [57]—an online platform that allows users to create “crowdsourcing tasks”, called Human-Intelligence Tasks (HITs), to be completed for small incentives. The Amazon MTurk platform allows anyone to become a requester in order to create HITs, while people that perform those tasks are called workers [58]. Recent studies that used Amazon MTurk have shown that it is an efficient, reliable, and cost-effective research tool for generating sample responses where human feedback is essential [59, 60].

**Fig. 2:**
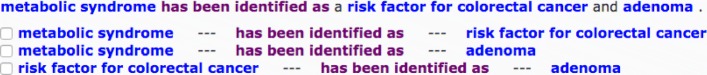
An example question of the crowdsourcing task. The terms in blue are biomedical entities, and terms in purple are the predicates describing the relations

We conducted two rounds of the crowdsourcing experiment. First, a pilot study was done in September, 2017 with a small sample (i.e., one HIT that contains 10 sentences) to establish the feasibility of using crowdsourcing workers to validate the candidate triples. The small sample was randomly selected from the corpus that we used to develop the original OC-2-KB system. All candidate triples for the 10 sentences were annotated by two members of the study team (the percent agreement was 80.41%). As shown in Fig. 2, each item of the HIT was a multiple choice question, where the question represented a sentence, and each option was a candidate triple statement describing the potential relationship between two biomedical entities and a predicate, as “*biomedical entity 1* — *relation* —- *biomedical entity 2*”, extracted from the sentence. Workers were asked to select the most appropriate triples statements that they thought were part of the sentence.

After the pilot study, in October 2017, we launched a bigger experiment with a data set of 82 abstracts from PubMed based on the same search keywords “obesity” and “cancer”. Two additional criteria were used to select the 82 abstracts: (1) articles that were review articles; and (2) articles that were published in reputable journals based on their impact factors published by Thomson Reuters. The 82 abstracts generated 671 sentences and 9,406 candidate triples. This data set is publicly available at https://github.com/bianjiang/BioText-2-KB/.

Based on experience from the pilot study, each MTurk HIT only contained 25 sentences. For each sentence, only at most the top 4 entities and top 3 predicates were extracted based on the *LIDF-value* [52] measure. We then used all possible combinations of the extracted entities and predicates as choices (i.e., candidate triples) to be validated by the workers. In sum, we created 27 HITs. Each question could be answered by 5 workers at most.

### Evaluations

We performed several experiments to (1) evaluate the crowdsourcing results, (2) used the crowdsourced triples to retrain the machine learning models considering a variety of crowdsourcing parameters (e.g., the number of times each triple was validated and how long workers spent on the HITs), and (3) evaluate the KBs created with- and without- the crowdsourcing feedback.

## Results

### The crowdsourcing experiments

#### The pilot study

The pilot study was carried out in eight days, from August 31, 2017 to September 7, 2017. We created only one HIT that contained 10 sentences, and the 10 sentences were annotated by two members of the study team. The number of assignments for the HIT was set to be 400 on MTurk, i.e., 400 workers could participate at most. The reward per assignment was set to be $0.15. The allotted time for the assignment was 900 s (i.e., the worker can hold to the task for a maximum of 900 s). A total of 193 workers participated in the pilot study. Table 1 shows the configuration and cost of the MTurk HIT for the pilot study.

**Table 1:** Configurations of the pilot crowdsourcing study

Data	Number of assignments per HIT	400
	Reward per assignment	$0.15
Estimated	Total reward	$60.0 (=400×*$*0.15)
	Fees to Mechanical Turk	$24.0 (=400×*$*0.6)
	Total cost	$84.0
Actual Cost	Assignments done and approved	193
	Total reward	$28.95
	Fees to Mechanical Turk	$11.58
	Total cost	$40.53

We also evaluated the time spent by the workers on the HIT, as shown in Table 2. We then evaluated worker performance in comparison to the gold-standard annotated by the experts, varying the amount of time spent by the workers. Table 3 shows the *F-measure* of each sentence.

**Table 2:** The time spent by the workers on the HIT

	Time spent on the HIT		
Time	≥ 0 s	≥ 120 s	≥ 300 s
Minimum (min)	38	127	302
Maximum (max)	889	889	889
Average ($\overline {x}$)	358.47	372.62	466.59
Standard Deviation (*σ*)	176.13	167.93	140.73

**Table 3:** Worker performance (F-measures) on the 10 sentences of the HIT

	Time spent on the HIT		
Sentences	≥ 0 s	≥ 120 s	≥ 300 s
Sentence 1	59.33%	60.24%	65.43%
Sentence 2	73.63%	75.03%	79.62%
Sentence 3	63.86%	65.30%	67.10%
Sentence 4	84.97%	84.78%	87.83%
Sentence 5	79.79%	80.62%	83.04%
Sentence 6	98.45%	98.37%	100.00%
Sentence 7	72.99%	74.02%	76.82%
Sentence 8	70.99%	72.67%	76.99%
Sentence 9	43.10%	43.30%	47.31%
Sentence 10	72.11%	73.54%	77.60%

Table 4 illustrates the overall worker performance in the pilot study (i.e., the average *F-measure (F)* of 10 sentences), varying by the time spent on the task. The best overall *F-measure* was 76.17%, when we accepted only answerers from workers that spent at least 300 seconds in the HIT.

**Table 4:** Overall worker performance in the pilot study

Time spent	Number of workers	F
≥ 0 s (0 s per sentence)	193	71.92%
≥ 120 s (12 s per sentence)	184	72.79%
≥ 300 s (30 s per sentence)	115	76.17 %

#### The final study

For the final study, we made our HITs available on MTurk for ten days, from October 12, 2017 to October 22, 2017. Our data set was 82 abstracts extracted from PubMed. These abstracts generated 671 sentences. The maximum number of entities and predicates extracted per sentence were 21 and 11, respectively. The maximum number of possible triples was 840 (i.e., obtained from a sentence with 16 entities and 7 predicates). As crowdsourcing tasks are typically micro-tasks [61], we selected the 4 most important entities and the 3 most important predicates for each sentence according to the *LIDF-value* [52], which yielded at most 18 triples per sentence. There were a total of 9406 possible triples extracted from the 671 sentences. We then created 27 MTurk HITs, where 26 HITs contained 25 sentences and 1 HIT contained 21 sentences (a total of 671 sentences). Each HIT was set to be completed by 5 workers at most (5 assignments). The reward per assignment was $0.5. The maximal allotted time for each assignment was 35 min (2100 s). Table 5 shows the configuration and cost of the 27 MTurk HITs. A total of 101 unique workers participated and completed 135 assignments (27 HITs × 5 assignments). As shown in Table 6, 89 workers completed only one HIT, while there is one worker completed 8 HITs.

**Table 5:** Configuration and price information of the final study

Data	Number of HITs	27
	Number of assignments per HIT	5
	Reward per assignment	$0.5
Estimated Cost	Total reward per HIT	$2.5 (=5×*$*0.5)
	Fees to Mechanical Turk per HIT	$0.5 (=5×*$*0.1)
	Total cost per HIT	$3.0 (=*$*2.5+*$*0.5)
	Total cost for 27 HITs	$81.0 (=*$*27×*$*3.0)
Actual Cost	Assignments done and approved	135 (=27 HITS ×
		5 assignments)
	Total cost	$81.0

**Table 6:** The number of HITs completed by the workers

Number of HITs	Number of workers that completed
1 HIT	89 workers
2 HITs	5 workers
3 HITs	1 worker
4 HITs	3 workers
5 HITs	0 worker
6 HITs	1 worker
7 HITs	1 worker
8 HITs	1 worker

We also evaluated the time spent by the workers, as shown in Table 7.

**Table 7:** Time spent by the workers over the 27 HITs

	Time spent on HITs		
Time	≥ 0 s	≥ 300 s	≥ 750 s
min	43	332	761
max	2095	2095	2095
$\overline {x}$	1,001.21	1,255.35	1,421.11
*σ*	638.27	495.58	382.54
Number of assignments completed ^∗^	5734	4413	3770

^*^The number assignments completed within the time range. Note that, there were 27 HITs, and each HIT was completed by 5 workers. Thus, there are a total of 135 assignments (27 HITs times 5 workers)

**Fig. 3:**
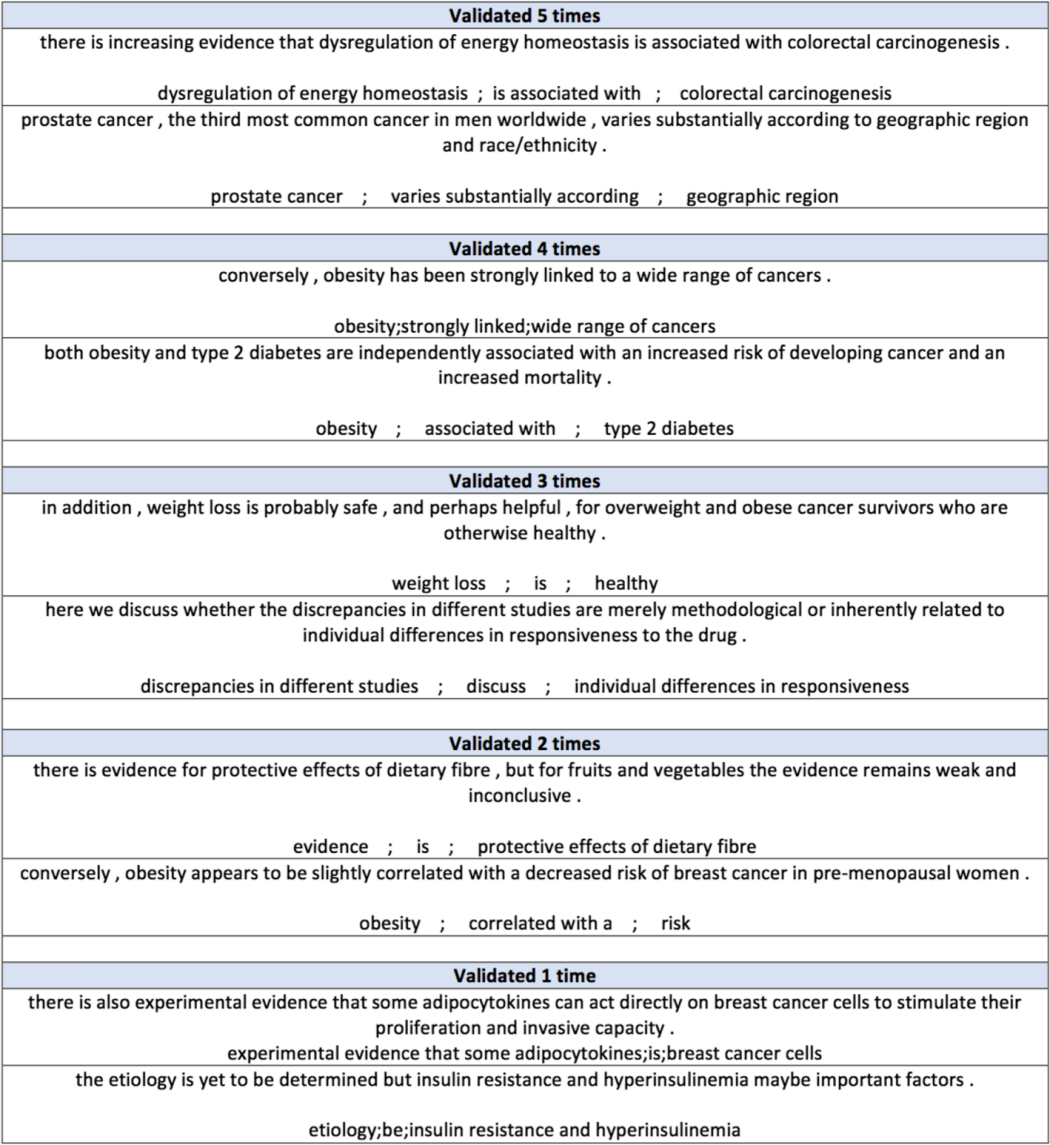
An example of triples validated through crowdsourcing

In addition to consider the different amount of time spent by the workers, we also considered the number of workers who made the same choices. Out of the 9406 possible triples, 3672 triples were not selected by any workers (i.e., none of the work who worked on the corresponding HIT considered the triple as valid). Table 8 presents the number of triples validated by more than 3, 4, and 5 workers (times) considering the different amount of time spent by the workers. Figure 3 shows an example of triples validated by different number of workers (i.e., more than 1, 2, 3, 4, and 5 times).

**Table 8:** Number of triples validated more than 3, 4, and 5 times varying by the workers’ time spent on the HITs

	Time spent on HITs		
The number of times validated	≥ 0 s	≥ 300 s	≥ 750 s
Validated = 5 times	37	19	15
Validated ≥ 4 times	258	109	68
Validated ≥ 3 times	918	506	320

**Fig. 4:**
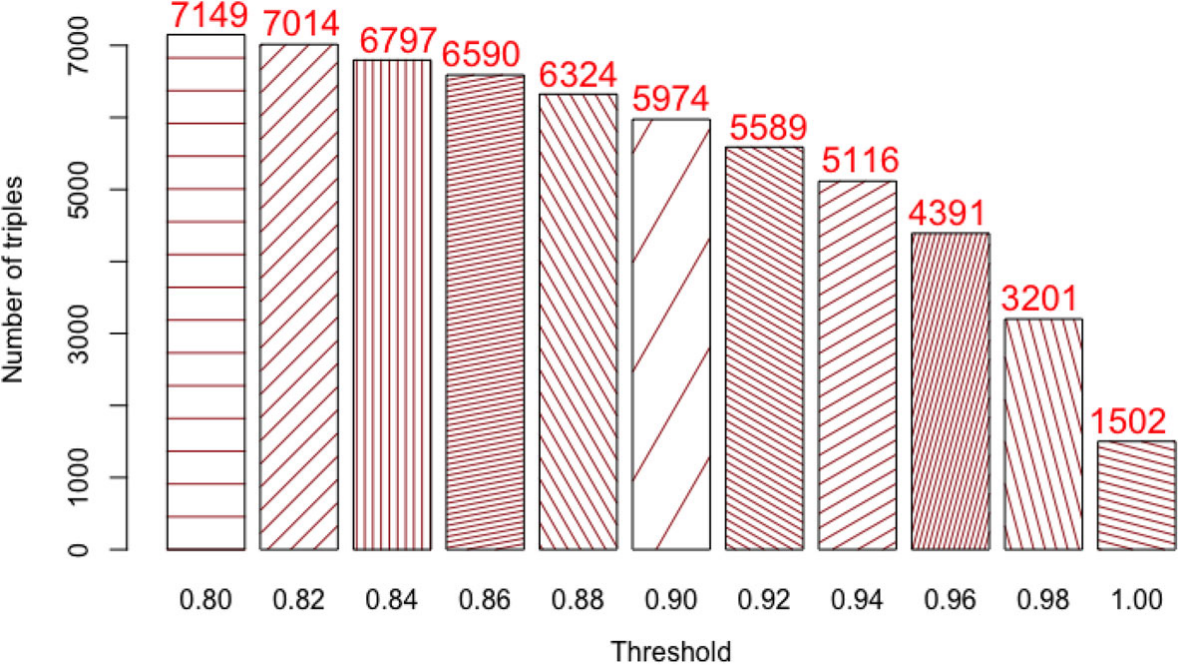
The number of triples created with the baseline OC-2-KB system varying the threshold *λ* from 0.80 to 1.00

We also used the baseline OC-2-KB system (i.e., where the relation detection machine learning model was trained using the initial 23 annotated abstracts) to extract triples from the 82 abstracts *(note that the baseline OC-2-KB system extracted 765 from the 23 abstracts and 4,391 from the 82 abstracts for which only 29% of the initial facts overlapped).* We considered different confidence scores (i.e., the probability that a triple is predicted to be true by the model). Figure 4 shows the number of triples extracted by the baseline OC-2-KB system varying the confidence score (threshold *λ* from 0.80 to 1.00) of the relation detection machine learning model.

**Fig. 5:**
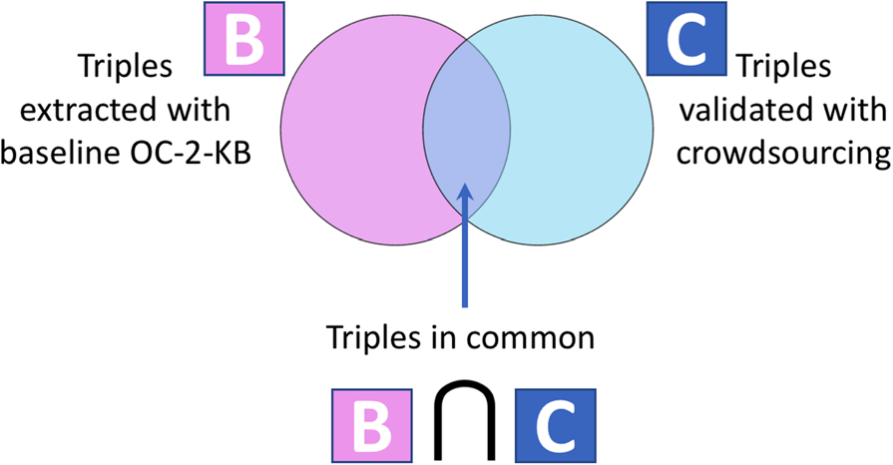
A Venn diagram of comparing the number of triples extracted with the baseline system (*B*) and validated through crowdsourcing (*C*), where the number of common triples between *B* and *C* is B ∩ C

We compared the predicted results obtained from the baseline OC-2-KB system with the crowdsourcing results for the same 82 abstracts, as shown in Fig. 5. In this comparison, we took into account the 918 triples that were validated by at least 3 workers without the time spent constraint. Table 9 shows the number of common triples between the baseline OC-2-KB system and the crowdsourcing results (B ∩ C), as well as the number of triples missed by the baseline OC-2-KB (C − B). The lowest rate of triples missed by the baseline system was 75.4% $\big (= \frac {692}{918}\big)$ with a low threshold (*λ*=0.80), while the highest rate of missed triples was 92.3% with a *λ*=1.00. As shown in Table 9, the baseline system missed a large number of triples, proving the necessity to augment the baseline model with human feedback.

**Table 9:** The number of common triples between the baseline OC-2-KB system and the crowdsourcing (B ∩ C), and the number of triples missed by the baseline OC-2-KB (C − B)

OC-2-KB _*λ*_	B ∩ C	C − B
*λ*=0.80	226	692
*λ*=0.82	222	696
*λ*=0.84	216	702
*λ*=0.86	215	703
*λ*=0.88	208	710
*λ*=0.90	198	720
*λ*=0.92	185	733
*λ*=0.94	171	749
*λ*=0.96	154	764
*λ*=0.98	119	799
*λ*=1.00	71	847

### Retraining of the relation detection model with crowdsourcing feedback

Leveraging the human feedback, we used the crowd-sourced annotations to improve the machine learning models in the baseline OC-2-KB system. We considered the different combinations of the crowdsourcing parameters (i.e., considering the different number of times each triple was validated, and the different amount of time spent by the workers) to extract the positive samples as shown in Table 8, and trained 9 relation detection models with the Random Forest algorithm. The random forest models achieved the best performance in our previous study for the same tasks [51]. We considered the triples that were not validated by any worker (i.e., 3672) as negative samples.

Our dataset was imbalanced, since we had significant more negative samples (i.e., 3672) than positive samples (i.e., ranging from 15 to 918, as shown in Table 8). Thus, we used weighted performance metrics to evaluate the trained machine learning models. Table 10 presents the weighted F-measures of the retrained RF models for relation detection considering the different crowdsourcing parameters.

**Table 10:** F-measures of the retrained random forest models varying the crowdsourcing parameters

	Workers’ time spent on HITs		
Number of times validated	≥ 0 s	≥ 300 s	≥ 750 s
Validated = 5 times	98.5%	98.8%	99.8%
Validated ≥ 4 times	90.3%	97.0%	97.2%
Validated ≥ 3 times	79.1%	85.1%	91.4%

### Evaluations of the knowledge bases created by the OC-2-KB system with and without the crowdsourcing feedback

To further assess the improvement of the retrained OC-2-KB system, we compared the KBs curated with the baseline OC-2-KB and retrained OC-2-KB (with the retrained model considering triples validated by more than 3 workers).

We assessed the content of the KBs (baseline OC-2-KB vs. retrained OC-2-KB) through evaluating whether the KB can answer specific competency questions. The competency questions were expressed in SPARQL queries. Note that the dataset in our original 23 abstracts was a balanced dataset (343 positive samples and 343 negative samples). To make a fair comparison, we randomly selected 918 negative samples from the 3672 triples that were not selected by any workers in the crowdsourcing experiment. In short, our training data for this part of the study were 1265 (347 + 918) positive samples and 1265 negative samples. The F-measure of the retrained relation detection model was 86.2% (ROCAUC: 0.935). For both the baseline and retrained OC-2-KB systems, we considered a threshold confidence score of 0.94.

Figure 7 shows an example of all the entities related to “breast cancer risk” (i.e., entities that can form the following assertions: <oc:breast cancer risk-?predicate-?object > or <?subject-?predicate-oc:breast cancer risk >). Note that in SPARQL, a query variable is marked by the use of “?”. Figure 7 (1) shows the associated query. Figure 7 (2) shows the query results from the KB created with the baseline OC-2-KB system. Figure 7 (3) illustrates the query results from the KB created with the retrained OC-2-KB system. Even though the results from the baseline contains more entities, the results from the retrained OC-2-KB are more accurate.

**Fig. 6:**
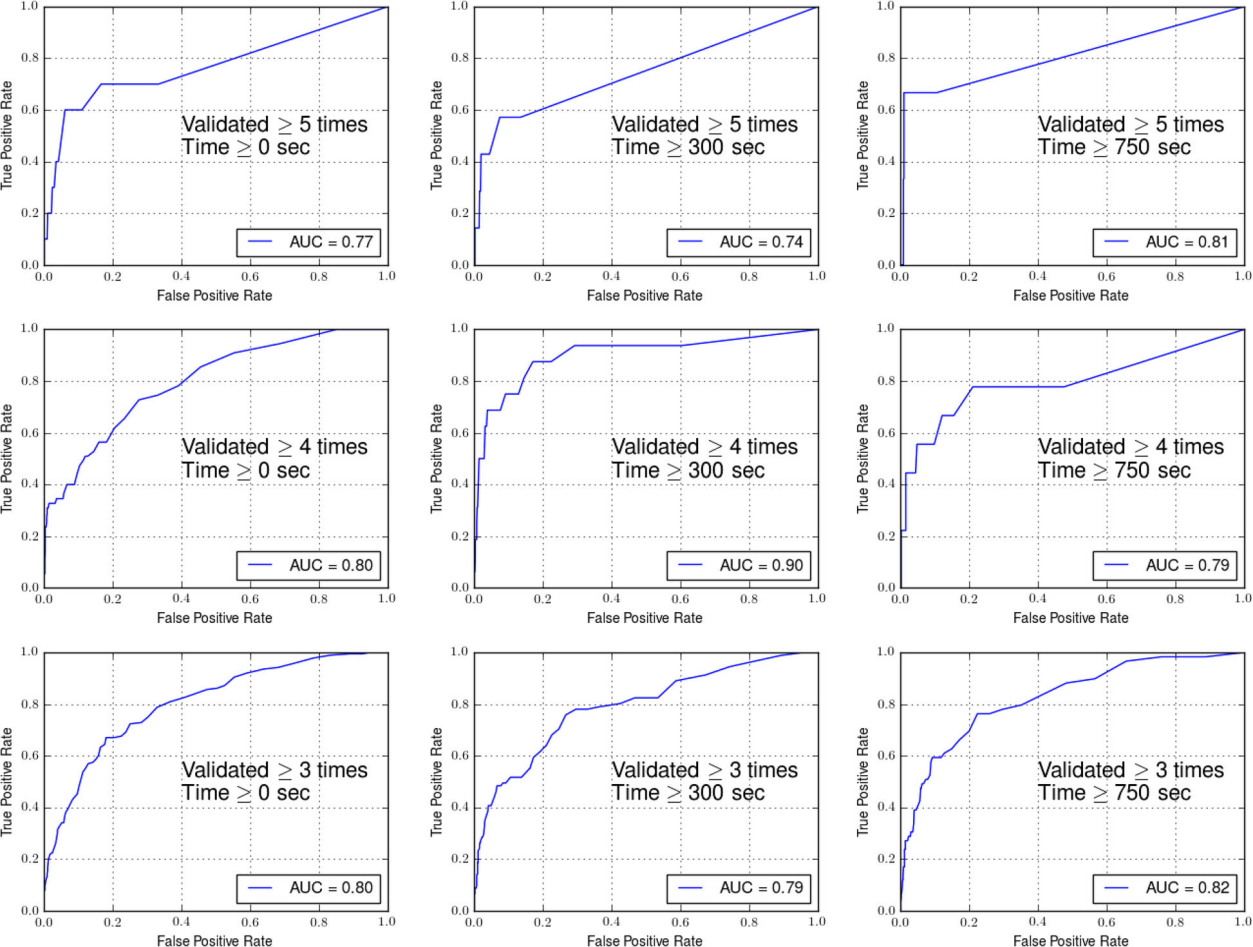
The receiver operating characteristic (ROC) curves of the retrained relation detection models

Figure 6 shows the receiver operating characteristic (ROC) curves of the retrained models.

## Discussion

### Feasibility and benefits of crowdsourcing

The non-expert crowdsourcing workers in the pilot study produced annotations comparable to the experts (i.e., an F-measure of 76.17*%*, which demonstrated the feasibility to use the crowd in annotation tasks. More importantly, even though our corpus was highly technical abstracts collected from scientific biomedical literature, with a careful design, cross-validated collective wisdom from laypeople can lead to quality knowledge collections. Thus, we used crowdsourcing to validate extractions that the machine identified as likely candidates. Further, validated extractions were then re-introduced into the training set for the machine learning models. As shown in Table 10 and Fig. 6, incorporating the crowdsourcing feedback significantly improved the performance of our machine learning models. Our approach is similar to the concept of active learning [62], a special case of semi-supervised machine learning where a learning algorithm is able to query the user to annotate new data points and incorporate the human feedback back into the machine learning model to improve its performance. However, as a pilot study, we selected new data to be labeled manually, while in a true active learning system, the system will be able to select unlabeled data and query the humans intelligently. This is indeed one of our future directions to create a more scalable KB construction framework.

Further, the costs of crowdsourcing studies are rather inexpensive, which is one of the other advantages of using crowd wisdom. Our pilot study received feedback from 193 workers and the cost was merely $40.53. Even if we only considered high quality responses (i.e., only accept answers from workers who spent more than 30 seconds on each sentence), 115 fell into this category. Further, the performance of these laypeople workers was comparable to expert annotators (i.e., an F-measure of 76.17*%*). In the final study, 89 workers completed 135 assignments (671 sentences) in 10 days, with a cost of $81. The low costs of crowdsourcing allowed us to collect redundant responses (i.e., each sentence was validated by 5 workers), which were then used to ensure the quality of the validated triples. Considering the majority rule, in our experiment, 918 triples were confirmed by at least 3 out of the 5 workers who worked on the same sentences.

The total cost of both studies on Amazon MTurk was $121.53. Amazon MTurk is proved to be an inexpensive way of gathering labeled annotations with human judgments.

### The quality of the created obesity and cancer knowledge base

**Fig. 7:**
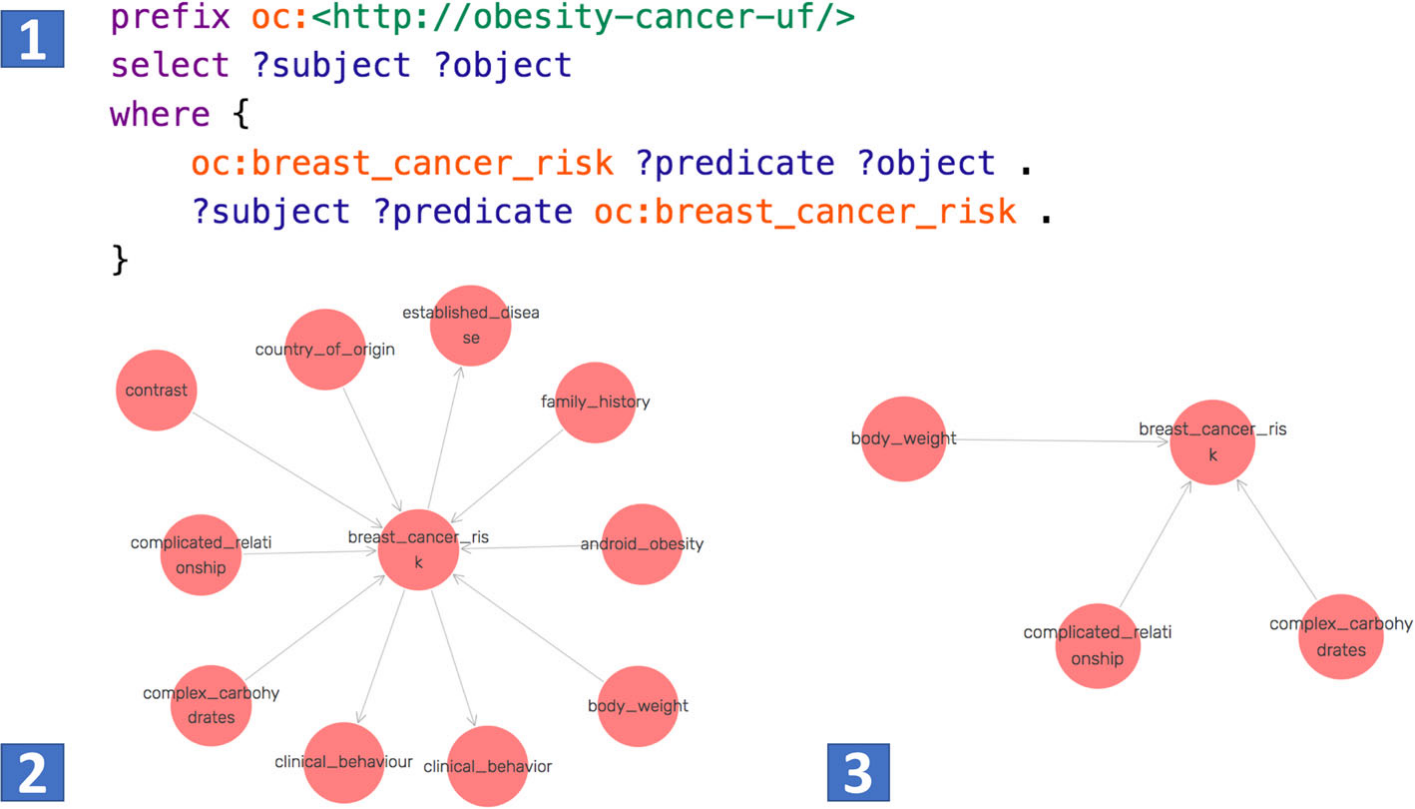
A SPARQL query for extracting all the entities related to “breast cancer risk” in (1). The results from the baseline OC-2-KB system are shown in (2), and the results from the retrained system are in (3)

There is a growing body of work on automated KB construction and knowledge extraction from text. Nevertheless, the value of these KBs lies in their ability to answer users’ questions (i.e., queries to the KBs). We thus evaluated the query results obtained from the OC-2-KB system with and without incorporating the crowdsourcing feedback. As shown in Fig. 7, the baseline OC-2-KB contained more but low-quality or unclear triples. For example, as shown in Fig. 7, the baseline OC-2-KB system incorrectly asserted a relation between “contrast” and “breast cancer risk”. On the other hand, the triples extracted by the retrained OC-2-KB system were of higher quality. However, it is also clear that the retrained OC-2-KB system missed some of the valid triples (e.g., the relation between “family history” and “breast cancer risks”), while eliminating more false positives.

**Fig. 8:**
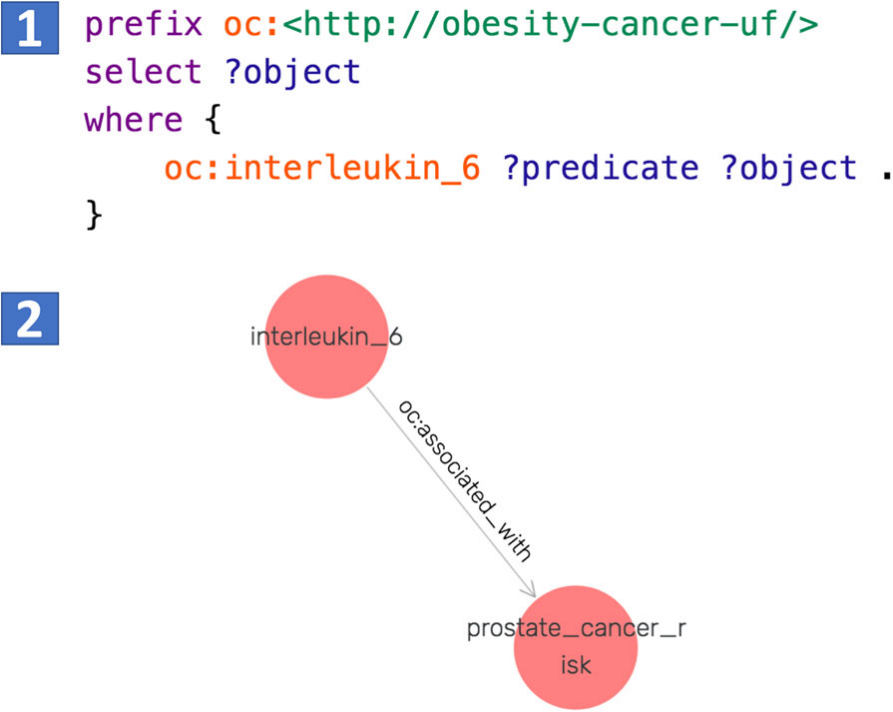
A SPARQL query for extracting entities related to “interleukin 6”

There were also triples identified by the retrained OC-2-KB system, but not by the baseline OC-2-KB system. Figure 8 shows a SPARQL query to extract any objects related to “interleukin 6”. The baseline OC-2-KB system did not return any results, while the retrained OC-2-KB extracted the assertion that <“interleukin 6”-“oc:associated with”-“prostate cancer risk” >.

**Fig. 9:**
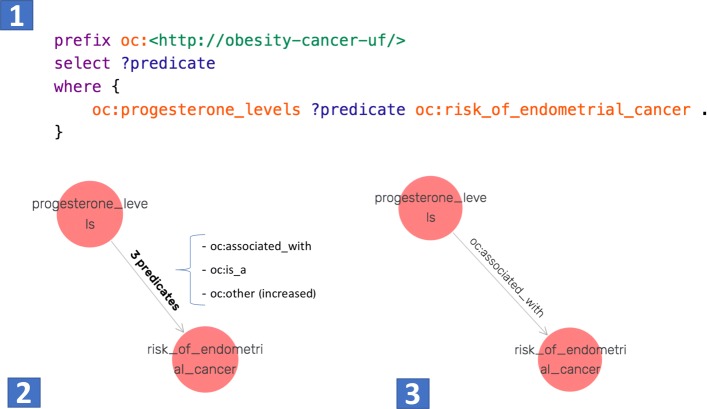
Results of all predicates existing between “progesterone levels” and “risk of endometrial cancer”

We also assessed the predicates of triples from both KBs (baseline vs retrained). We found that in most cases the KB created with the baseline OC-2-KB extracted either incorrect predicates or semantically similar but syntactically different predicates for the same two entities. For example, Fig. 9 shows a SPARQL query to identify all relations between “progesterone levels” and “risk of endometrial cancer”. The baseline OC-2-KB contained 3 predicates: “oc:associated with”, “oc:is a”, and “oc:other (increased)”, while the retrained OC-2-KB only contained one predicate “oc:associated with” between the same two entities.

**Fig. 10:**
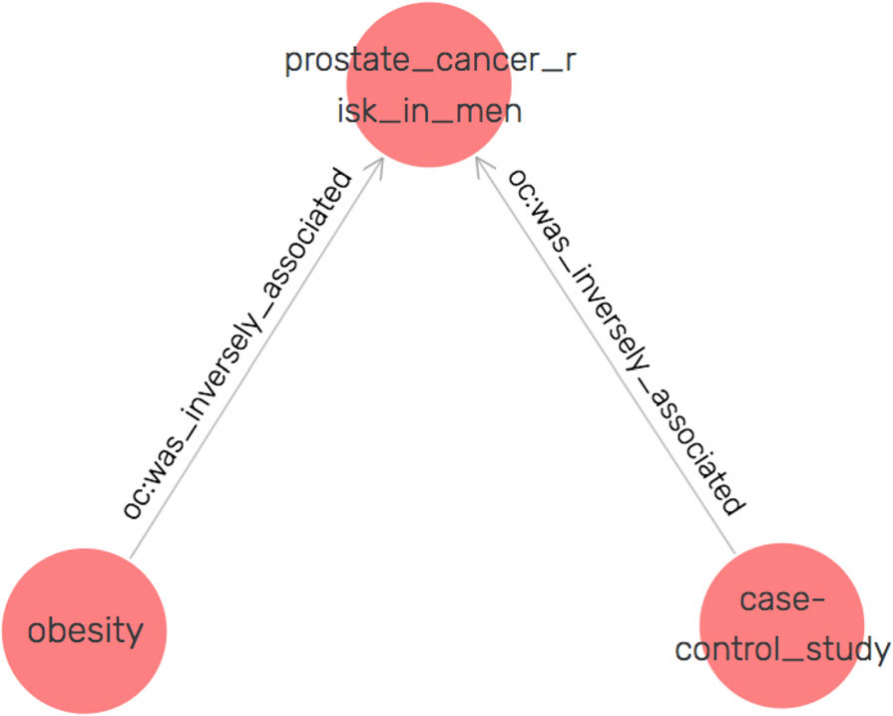
Example of invalid triples associated with “prostate cancer risk in men”

The curated KBs, although improved with crowdsourcing feedback, still contained invalid or not meaningful triples. This is because that the accuracy of our automated relation extraction methods although achieved state-of-the-art performance is still suboptimal. Figure 10 shows an example of invalid triples related to “prostate cancer risk in men’. From the original sentence, *(“Similarly, a case-control study found obesity was inversely associated with prostate cancer risk in men aged 40-64 years.”)*, OC-2-KB extracted <“case-control study” oc:was inversely associated “prostate cancer risk in men” >, which is incorrect. This emphasizes the needs to consider humans in the loop and to use crowd wisdom to validate triples identified by the machine.

## Conclusions

We presented a new version of OC-2-KB, a system that automatically builds an evidence-based obesity and cancer KB from scientific literature. We developed a scalable framework for the construction of the KB by integrating automatic information extraction with crowdsourcing techniques to verify the extracted knowledge.

Crowdsourcing platforms such as Amazon MTurk offers a low-cost mechanism to collect more labeled data from the crowd with non-expert laypeople. Even though individual layperson might not offer reliable answers, the collective wisdom of the crowd is comparable to expert opinions.

However, further studies are still warranted, as the performance of the underlying information extraction system in OC-2-KB is still suboptimal, possibly due to the noisy nature of free-text data (e.g., inconsistent formatting, alternative spellings, and misspells). We shall further investigate how to better leverage the crowdsourcing platforms. For example, we shall further explore the active learning concept, where 1) the triples that need to be validated by the crowd are identified by the machine learning model, and 2) both the precision and recall of the machine learning model will be improved with the crowdsourcing feedback, without losing existing valid triples.

The ultimate goal of this project is a paradigm shift in how the general public access, read, digest, and use online health information. Rather than requiring the laypeople find and read static documents on the Internet via regular searches, we propose a dynamic knowledge acquisition model, where the content is routinely mined from the scientific literature and vetted, users interact via semantic queries instead of regular searches, and consumers navigate the network of knowledge through interactive visualizations.

## Abbreviations

F: F-measure
IE: Information Extraction
ML: Machine Learning
NER: Named-entity Recognition
NLP: Natural Language Processing
OC-2-KB: Obesity and Cancer to Knowledge Base
OWL: Web Ontology Language
RDF: Resource Description Framework
RE: Relation Extraction
RF: Random Forest
RO: Relation Ontology
ROCAUC: Receiver operating characteristic area under curve
SPARQL: SPARQL Protocol and RDF Query Language
